# Laboratory Rearing of the Photosynthetic Sea Slug *Elysia crispata* (Gastropoda, Sacoglossa): Implications for the Study of Kleptoplasty and Species Conservation

**DOI:** 10.3390/biology15020168

**Published:** 2026-01-17

**Authors:** Paulo Cartaxana, Diana Lopes, Vesa Havurinne, Maria I. Silva, Ricardo Calado, Sónia Cruz

**Affiliations:** Laboratory for Innovation and Sustainability of Marine Biological Resources (ECOMARE), Centre for Environmental and Marine Studies (CESAM), Department of Biology, University of Aveiro, Campus Universitário de Santiago, 3810-193 Aveiro, Portugal; lopes.diana@ua.pt (D.L.); vesa.havu@ua.pt (V.H.); mifrsilva@ua.pt (M.I.S.); rjcalado@ua.pt (R.C.)

**Keywords:** *Acetabularia acetabulum*, *Bryopsis*, chloroplast, endosymbiosis, kleptoplast, marine aquarium trade

## Abstract

Some Sacoglossa sea slugs are capable of stealing and maintaining functional intracellular chloroplasts—kleptoplasts—from their macroalgal prey for periods of up to several months, a process known as kleptoplasty. Although the cultivation of these marine invertebrates under laboratory conditions is crucial for research in various fields (e.g., endosymbiosis, animal physiology, discovery of new marine natural products), rearing protocols are scarce. This study presents a standardized protocol for the laboratory rearing of large numbers of the sacoglossan tropical sea slug *Elysia crispata*. The detailed protocol successfully facilitated embryonic development, larval metamorphosis, and juvenile-to-adult transition, allowing the rearing of multiple generations. Two groups, characterized by acquiring different kleptoplasts, were obtained by feeding the sea slugs with two different prey macroalgae: *Bryopsis* sp. and *Acetabularia acetabulum*. Usually referred to as lettuce sea slug among marine aquarium hobbyists, *E. crispata* is a highly valued organism for its striking appearance and ability to control nuisance algal growth in reef aquariums. This protocol allows experimental reproducibility and access to specimens under different development stages, potentially boosting research on kleptoplasty while also contributing to reducing the impact of the marine aquarium trade on natural populations.

## Simple Summary

The sea slug *Elysia crispata* is commonly used in the study of kleptoplasty, a mechanism by which host cells can capture and retain long-term functional chloroplasts—kleptoplasts—from their algal prey. This extraordinary act of thievery has intrigued scientists for over a century. Moreover, *E. crispata* is commonly acquired by marine aquarium hobbyists because of its ability to eat nuisance macroalgae in reef aquariums. This study presents a detailed protocol for the successful rearing of multiple generations of *E. crispata* and characterizes two groups retaining kleptoplasts from two different prey macroalgae. This opens new options in experimentally addressing the kleptoplasty conundrum not possible using sea slugs collected from the wild, while also potentially contributing to species conservation.

## 1. Introduction

Heterobranch sea slugs (Gastropoda, Heterobranchia) comprise a highly diversified and successful group of marine gastropod mollusks that present a global distribution and occupy a wide range of ecological niches, garnering attention from both the scientific community and the general public [1]. Sea hares, *Aplysia* spp., have become pivotal model organisms in neurobiology due to their relatively simple nervous systems and large, easily accessible neurons [2]. Nudibranchs have gained prominence in neuroethological studies, particularly in elucidating the neural bases of complex behaviors [3], as well as in biotechnology due to their potential as sources of new marine natural products [4]. Members of the Sacoglossa, a clade of herbivorous marine heterobranchs, can be used as potential model organisms in the study of the early stages of endosymbiosis that led to organelle establishment because of their capacity to steal and maintain functional chloroplasts—kleptoplasts—from their macroalgae prey [5]. Within the Metazoa, the long-term (weeks to months) retention of functional chloroplasts is unique to Sacoglossa, contributing to sea slug energy metabolism, reproduction, and defense mechanisms [6].

To facilitate comprehensive studies on kleptoplasty and the evolutionary incorporation of intracellular symbionts into organelles, the development and optimization of laboratory culture protocols are imperative [7]. Ex situ cultivation provides enhanced control over population dynamics and environmental parameters without compromising natural populations. Access to organisms across all development stages is crucial to experimentally address specific scientific questions. Furthermore, experiments with lab-reared specimens offer superior control over confounding variables, such as age, fitness, dietary history, and acclimation status. Importantly, refined culture protocols reduce dependence on wild specimens, thereby contributing to the conservation of vulnerable species naturally occurring at low densities [8].

*Elysia crispata* Mörch, 1863, popularly known as lettuce sea slug, is among the largest known marine sacoglossans, primarily distributed in the shallow tropical waters of the Caribbean Sea and Gulf of Mexico [9]. The species shows pronounced color and pattern polymorphism and variable external morphology (e.g., parapodial frilling, spotting). A morphological and molecular distinction between *E. crispata* and a Florida Keys population was proposed by Pierce et al. [10], who described the latter as a new species, *Elysia clarki*. A subsequent integrative taxonomic revision [9] found no consistent diagnostic differences and synonymized *E. clarki* with *E. crispata*, a view followed by most recent studies [11,12].

*Elysia crispata* exhibits simultaneous hermaphroditism, with each individual possessing both male and female gonads and reciprocally exchanging sperm between sexual partners. The species shows obligatory primary lecithotrophic larval development, a reproductive strategy characterized by developing organisms relying exclusively on maternally provisioned yolk reserves to reach metamorphosis, rather than on exogenous feeding [9]. This type of development confers several adaptive advantages, including a consistent nutrient supply that is key to successfully reaching metamorphosis [13]. Maternal yolk provides essential macromolecules and energy substrates for cellular proliferation and differentiation, enabling larvae to develop fully within protective egg capsules. Upon hatching, *E. crispata* offspring already emerge as imagos of adults that are able to immediately start feeding; this trait most likely enhances the survival of these organisms in their natural habitat [13]. While research on *E. crispata* has been relatively limited compared to other heterobranch taxa, there is growing interest in its potential as a model organism, for example, in the study of kleptoplasty [7] and as a source of bioactive compounds [14].

*Elysia crispata* is a polyphagous species, meaning it may feed on a wide variety of macroalgae in the wild. Polymerase chain reaction (PCR)-based DNA barcoding has identified an enormous inter-population variability in the algal source of *E. crispata* kleptoplasts, mostly originating from *Bryopsis*, *Halimeda* and *Penicillus* species [15,16,17]. However, its algal food preference is highly dependent on the development stage, with newly metamorphosed juveniles feeding on and sequestering chloroplasts from algal species different from those utilized by adult sea slugs [18]. In the latter study, recently hatched juveniles were only able to feed on the thinner filamentous algae *Bryopsis plumosa* and *Derbesia tenuissima* out of a set of 29 algal species, and the authors hypothesized that the very fine filaments of the two algae could be easier for the juvenile sea slugs to grasp and perforate. Algae of the genus *Acetabularia* have also been reported as a kleptoplast source of *E. crispata*, although suggested as a low-preference food source [15,19,20]. However, in this sea slug species, a significantly longer longevity was observed for *Acetabularia acetabulum*-derived kleptoplasts when compared to *Bryopsis* [21]. Furthermore, significantly lower relative weight loss under starvation was observed for *E. crispata* previously fed on *A. acetabulum* than on *Bryopsis* [21].

The present work describes a standardized protocol for the laboratory rearing of large numbers of *E. crispata* under controlled conditions and characterizes two different groups of cultured specimens that harbor diverse kleptoplasts, obtained by feeding upon two different prey macroalgae: *Bryopsis* sp. and *A. acetabulum*.

## 2. Materials and Methods

### 2.1. Animal Acclimatation and Maintenance

**Figure 1:**
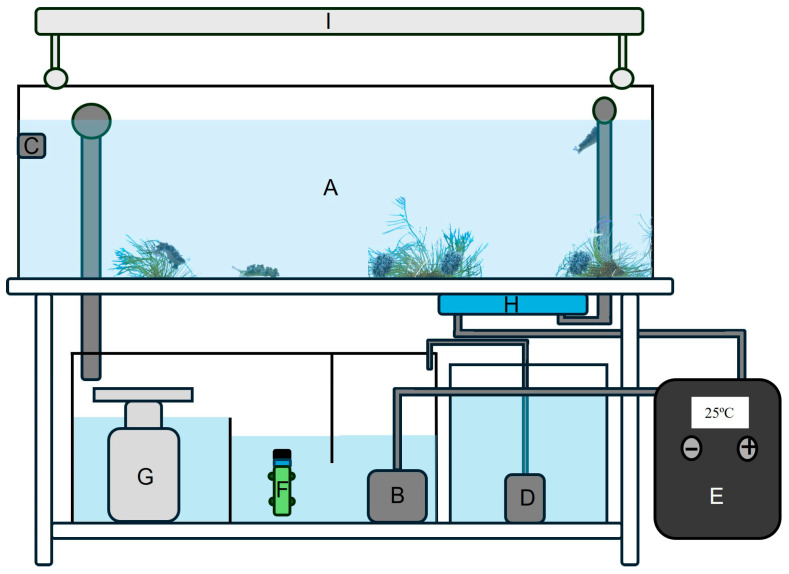
Schematic representation of the life-support system (LSS) used to house *Elysia crispata*. (**A**) Main aquarium with sea slugs and macroalgae; (**B**) recirculation water pump; (**C**) powerhead; (**D**) osmoregulator; (**E**) chiller; (**F**) heater; (**G**) protein skimmer; (**H**) UV sterilizer; (**I**) lighting system. Not to scale.

Twenty adult specimens of the tropical sacoglossan sea slug *Elysia crispata*, collected in Florida, were acquired from a marine aquarium wholesale supplier (Tropical Marine Centre Iberia, Lisbon, Portugal) and maintained under controlled laboratory conditions. All specimens were kept in a recirculated life-support system (LSS) with a total volume of approximately 200 L and operated using artificial seawater (ASW) at a salinity of 35 (Figure 1) [21]. The ASW was prepared by dissolving a commercial mix of salts (Red Sea Europe, Verneuil d’Avre et d’Iton, France) in freshwater purified by reverse osmosis (MO12000 E; Ambietel—Tecnologias Ambientais LDA., Matosinhos, Portugal). The LSS included the main aquarium with the sea slugs and a water recirculating pump securing a flow of 1200 L h^−1^ (Universal 1250; Eheim GmbH & Co. KG, Deizisau, Germany) housed in a 75 L reservoir located under the main aquarium (Figure 1A,B). To ensure a suitable water circulation within the main aquarium, a powerhead (Turbelle nanostream 6015; Tunze, Penzberg, Germany) was placed in the main aquarium securing a water flow of up to 1800 L h^−1^ (Figure 1C). A mesh screen was used to protect the sea slugs from the power head propeller. The LSS also contained an osmoregulator (Aquastat 1001; Deltec GmbH, Delmenhorst, Germany), which was connected to a submerged pump (Compact 1001, Eheim GmbH & Co. KG, Deizisau, Germany) placed in a reservoir filled with freshwater purified by reverse osmosis to compensate for evaporation (Figure 1D). The combined use of a HC-150A chiller (Hailea; Chaozhou, China) and a 300 W heater (Eheim Jäger; Eheim GmbH & Co.KG, Deizisau, Germany) allowed for a precise control of water temperature in the system at 25 ± 1 °C (Figure 1E,F). A protein skimmer (SC 1455; Deltec GmbH, Delmenhorst, Germany) was used to remove organic waste from the water and a 25 W UV filter (Vecton 600; Tropical Marine Centre Ltd., Hertfordshire, UK) was employed to reduce the load of harmful microorganisms to guarantee suitable water quality (Figure 1G,H).

Regular maintenance activities included the weekly cleaning of the main aquarium and additional reservoir, with partial water changes of about 10% of the total water volume of the LSS also being performed. The levels of ammonium, nitrates and phosphates (<0.02, <0.5 and <0.05 mg/L, respectively) were monitored weekly using colorimetric tests (Tropical Marin, Wartenberg, Germany). Every three weeks, a thorough cleaning of all LSS components was performed, as well as a renewal of about 20% of the total water volume of the LSS. The LSS was equipped with either T5 fluorescent lamps (54 W T5 HO; Hailea, Chaozhou, China) or LED aquarium lights (V165, ViparSpectra, Richmond, CA, USA) providing a photon scalar irradiance of 60–80 μmol photons m^−2^ s^−1^ (Figure 1I). Light was measured at the water surface using a spherical quantum sensor connected to ULM-500 Universal Light Meter (Heinz Walz GmbH, Effeltrich, Germany). A photoperiod of 12 h–12 h light–dark was maintained using a digital timer. Specimens of *E. crispata* were fed with either the macroalgae *Bryopsis* sp. or *Acetabularia acetabulum* (see below).

### 2.2. Algae Culturing

The *Bryopsis* sp. strain KU-0990 was obtained from Kobe University Macroalgal Culture Collection (Japan) in 2018 and maintained in culture ever since. Algal cultivation was performed in round-bottom flasks containing 2 L of ASW enriched with f/2 medium (minus silica), at 20 °C under constant aeration. Illumination for macroalgal cultures was provided by LED lamps (Valoya 35 W, spectrum NS12) with an irradiance of 60–80 μmol photons m^−2^ s^−1^ and a photoperiod of 12 h–12 h light–dark secured by a digital timer. The green alga *A. acetabulum* (strain DI1 originally isolated by Diedrik Menzel) was cultivated in 11 L rectangular transparent plastic boxes (395 × 245 × 130 mm) filled with f/2 medium without aeration. *A. acetabulum* was cultured without aeration to avoid contamination that would ultimately lead to the loss of the entire culture. The photon scalar irradiance was set to 40 μmol photons m^−2^ s^−1^ in a 12 h–12 h light–dark photoperiod. The protocol described by Hunt and Mandoli [22] was successfully employed to secure the sexual reproduction of *A. acetabulum*.

### 2.3. Spawning and Larval Development

After one month under the laboratory conditions described above, multiple egg masses started being observed in different substrata within the LSS; these included the glass surfaces of the main tank, lithic structures, meshes, and even the macroalgae being supplied as food. To ensure structural integrity, egg masses were left in situ before collection for 24 to 48 h post-oviposition. This delay in collection allowed for increased mechanical stability of the egg masses, facilitating their removal using a scalpel blade. Collected egg masses underwent a decontamination process involving gentle washing with autoclaved ASW at a salinity of 35 and 25 °C. This procedure was crucial for the removal of potential contaminants and debris. Sterile tweezers were used to manually excise any adhering *Bryopsis* sp. filaments, and each egg mass was transferred to individual sterile 250 mL Erlenmeyer flasks containing about 200 mL of autoclaved ASW at the aforementioned salinity and temperature (see Section 4 for a suggested alternative). The Erlenmeyer flasks were maintained in a temperature-controlled incubator (Cooled Incubator MIR-154; Panasonic, Osaka, Japan) at 25 °C. Illumination was provided at a photon scalar irradiance of 60 μmol photons m^−2^ s^−1^, with a photoperiod of 12 h–12 h light–dark. To maintain optimal chemical parameters, partial water changes (50% of total volume) were performed at 48 h intervals. Aeration was not used in this step, as it contributed to a degradation of the egg masses, leading to the loss of most, if not all, offspring. During the initial 14 days post-oviposition, the egg masses exhibited a gradual transition from a white to a gray tone, indicative of larval development.

### 2.4. Juvenile to Adult Transition

Upon hatching, juveniles were collected using a 50 μm mesh sieve and subsequently transferred to sterile 250 mL Erlenmeyer flasks containing fresh *Bryopsis* sp. This collection and transfer protocol was implemented every two days until all offspring had emerged from the egg mass. Partial water exchanges (50% of total volume) were performed concurrently with the collection of recently hatched organisms. Continuous aeration was supplied to ensure adequate gas exchange. When supplied with *A. acetabulum*, all the newly hatched juveniles were unable to feed upon this macroalga and died.

When the juveniles attained visible size to the unaided eye (approximately 0.5 mm in total length), they were transferred to 5 L rectangular plastic containers (245 × 155 × 130 mm) with approximately 3 L of water from the LSS to which they would eventually be transferred and provisioned with *Bryopsis* sp. This intermediate step facilitated gradual acclimation to the LSS conditions. Upon reaching a total length of approximately 2 mm, at which point juveniles already display morphological features analogous to adults, they were introduced into the LSS.

### 2.5. Group Characterization

Newly hatched juvenile *E. crispata* were unable to feed upon *A. acetabulum*. However, sea slugs that were approximately 3 months old and were fed on *Bryopsis* sp. were able to readily shift their diet when offered *A. acetabulum* (as previously observed by Cartaxana et al. [21]). Sea slugs completely changed their appearance, from a dark to a much lighter green, within 5 to 10 days of this diet shift. To confirm if sea slug color changed as a result of kleptoplast replacement, a pigment characterization of two groups of sea slugs, one fed on *Bryopsis* sp. and another fed on *A. acetabulum* was performed using high performance liquid chromatography (HPLC) analysis. Briefly, five sea slugs were flash frozen in liquid nitrogen and freeze-dried. Approximately 4 mg of dry weight were extracted for 20 min at –20 °C in 1 mL 95% cold buffered methanol (2% ammonium acetate), after 1 min sonication. After filtration through 0.2 μm Fisherbrand™ PTFE membrane filters, the obtained extracts were immediately injected into a Prominence-i LC 2030C HPLC system (Shimadzu, Kyoto, Japan) equipped with a photodiode array detector. Chromatographic separation was carried out using a Supelcosil C18 column (dimensions: 250 × 4.6 mm; particle size: 5 μm; Sigma-Aldrich, St. Louis, MO, USA) for reverse phase chromatography. Photosynthetic pigments were identified from retention times and absorption spectra, in comparison with pure standards from DHI (Hørsolm, Denmark).

Kleptoplasts from *E. crispata* fed with either *Bryopsis* sp. or *A. acetabulum* were imaged using a confocal laser scanning microscope (CLSM), essentially as previously described by Havurinne et al. [23]. Briefly, five animals were decapitated swiftly with a razor blade, and a piece of the parapodia was excised and placed between a microscope slide and a cover glass. The images were acquired using a Mica Microhub (Leica Microsystems, Wetzlar, Germany) CLSM, using the HC PL APO 63×/1.20 W CS2 motCORR objective and the associated Leica Application Suite X (LASX, version 6.2.2.28360) software. Chlorophyll *a* excitation laser wavelength was set at 405 nm and the emission detection range was automatically determined by the software using chlorophyll *a* absorption and emission spectra (downloaded from https://www.fpbase.org/spectra/, last access on 14 January 2026). Image capture settings were optimized for each image individually using the “OneTouch” feature, and the “Lightning” computational clearing of the images was always on during image capture. A total of 30 kleptoplasts were segmented manually from the images in Fiji [24], and the areas of individual kleptoplasts were measured as an indicator of kleptoplast size.

## 3. Results

### 3.1. Life Cycle

**Figure 2:**
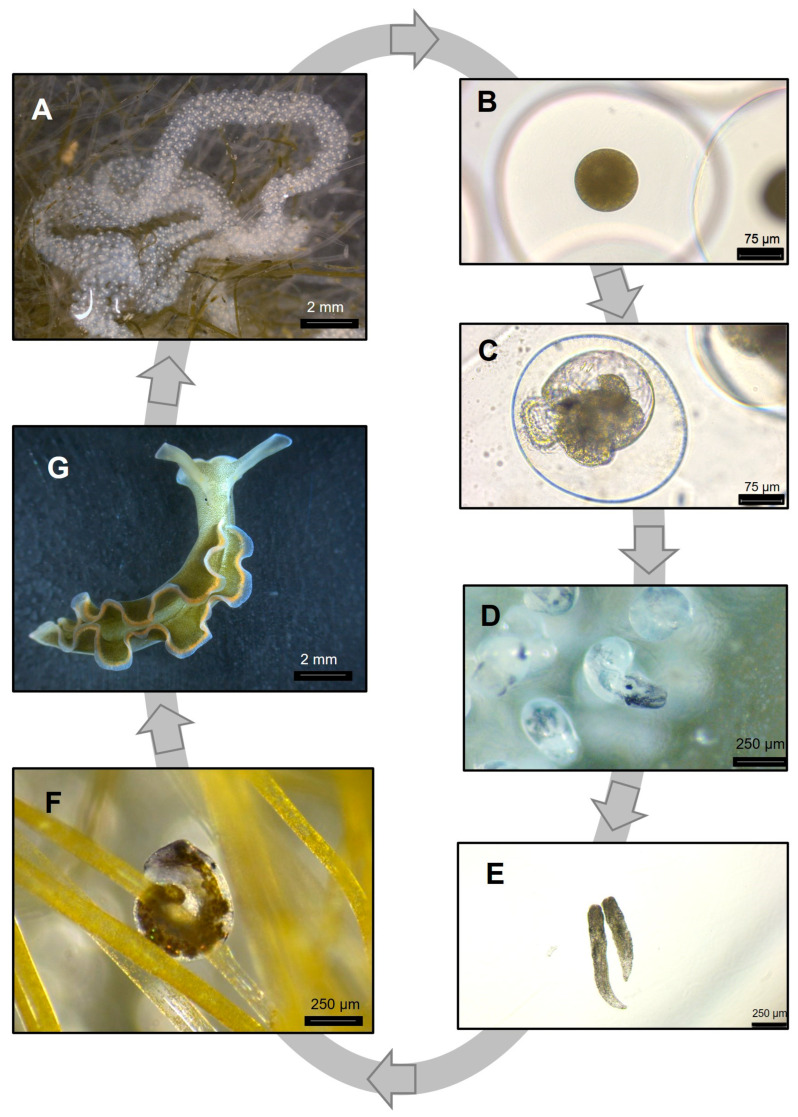
Life cycle of the sea slug *Elysia crispata*. (**A**) Egg string spawned around the filaments of *Bryopsis* sp.; (**B**) round-shaped egg immediately after spawning; (**C**) developing larva 8 days post-spawning; (**D**) hatching of recently metamorphosed juvenile; (**E**) recently hatched crawling sea slugs before feeding; (**F**) recently hatched sea slug feeding on the macroalga *Bryopsis* sp.; (**G**) 3 month-old sea slug. Photos were obtained with a digital microscope (DMS-300, Leica Microsystems, Wetzlar, Germany; (**A**,**D**,**F**,**G**)) or an inverted microscope (DMi1, Leica Microsystems, Wetzlar, Germany; (**B**,**C**,**E**)). See Supplementary Figure S1 for key operations in the different development stages.

Following a one-month acclimation period to laboratory conditions, adult *E. crispata* specimens readily formed breeding pairs and started spawning. The precise age of these wild-collected individuals was undetermined. Each spawned egg mass comprised up to three thousand embryos encapsulated in a gelatinous matrix (Figure 2A). Individual embryos had a perfect spherical structure upon spawning events (Figure 2B), but cell division initiated immediately after, within the first 24 h post-spawning. After the first week post-spawning, larvae exhibit well-defined morphological features (Figure 2C). In this initial period, larvae showed rotatory ciliary movements inside egg capsules. Larval development spanned approximately 14 days. Developing larvae underwent intracapsular metamorphosis at the end of this period and young juveniles moved vigorously in an attempt to hatch, while the egg capsule lost its original round shape (Figure 2D). In the usually spiral-shaped egg masses, it was possible to observe offspring in different developmental stages. Individuals hatched as crawling juveniles and immediately started feeding on *Bryopsis* sp., swiftly leading to the appearance of a dark green coloration (Figure 2E,F). Small juveniles were observed firmly grasping the fine algal filaments of *Bryopsis* sp. When juveniles started to exhibit adult morphological features, such as rhinophores and rippled parapodia (Figure 2G), they continued their development in an LSS, sustained by regular macroalgal consumption. Individuals reaching an age of approximately 6 months started to mature sexually and reproduce, regularly spawning new egg masses. Sea slugs reared under these laboratory conditions lived, in general, for about one year.

### 3.2. Kleptoplast Origin

**Figure 3:**
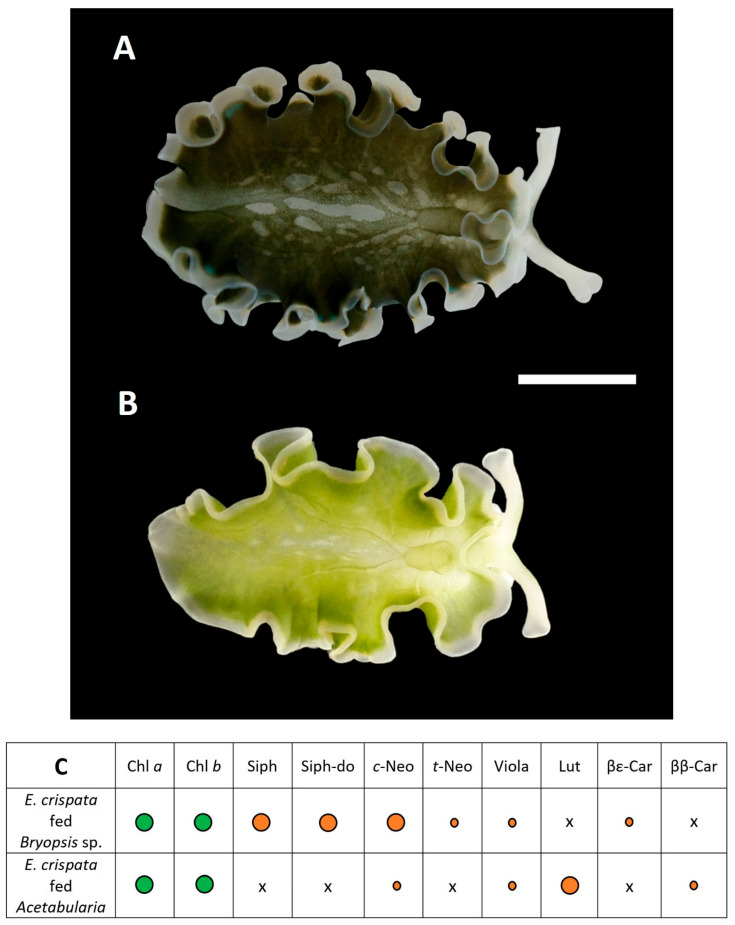
*Elysia crispata* sea slugs harboring different kleptoplasts. (**A**) The dark green adult specimen of *E. crispata* fed on the macroalga *Bryopsis* sp.; (**B**) the light green adult specimen of *E. crispata* fed on the macroalga *Acetabularia acetabulum*; (**C**) pigment composition of the sea slugs harboring the different kleptoplasts. O—major pigment; o—minor pigment; x—absent pigment. Chlorophylls in green and carotenoids in orange. Chl *a*: chlorophyll *a*; Chl *b*: chlorophyll *b*; Siph: siphonaxanthin; Siph-do: siphonaxanthin dodecenoate; *c*-Neo: *cis*-neoxanthin; *t*-Neo: *trans*-neoxanthin; Viola: violaxanthin; Lut: lutein; βε-Car: β,ε-carotene; ββ-Car: β,β-carotene. Scale bar represents 10 mm. Sea slugs were anesthetized with clove oil as described by Cruz et al. [25] to capture the open parapodia. Photos were obtained with a Canon EOS 450D digital camera (Canon, Tokyo, Japan).

Sea slugs of approximately 3 months fed on *Bryopsis* sp. were able to shift their diet when offered *A. acetabulum*. These sea slugs developed normally, attained sexual maturation and reproduced. Hence, it was possible to establish two distinct groups of cultured *E. crispata*, one exclusively fed on *Bryopsis* sp. throughout their life span (Figure 3A), and another one where diet was first composed of *Bryopsis* sp. but subsequently shifted to *A. acetabulum* (Figure 3B). The specimens of these two groups showed completely different body colorations.

To ensure that these two different groups harbored kleptoplasts exclusively from one of these macroalgae, a detailed pigment characterization was performed. Sea slugs from both groups shared chlorophylls *a* and *b*, as well as the xanthophylls *cis*-neoxanthin and violaxanthin (Figure 3C; Supplementary Figure S2; Supplementary File S1). However, the carotenoid (xanthophylls + carotenes) profile was clearly distinct between the two groups. Sea slugs exclusively fed on *Bryopsis* sp. showed xanthophylls siphonaxanthin and siphonaxanthin dodecenoate (siphonein), *trans*-neoxanthin, and the carotene β,ε-carotene, with these pigments being characteristic of its prey macroalga. On the other side, sea slugs fed on *A. acetabulum* showed the presence of the xanthophyll lutein and β,β-carotene, which are characteristic of this macroalga. After just 10 days of feeding, no traces of *Bryopsis* sp. diagnostic pigments were found on this group of sea slugs that had been fed on their first three months of life with this macroalga, including none of its major xanthophylls (siphonaxanthin and siphonein).

**Figure 4:**
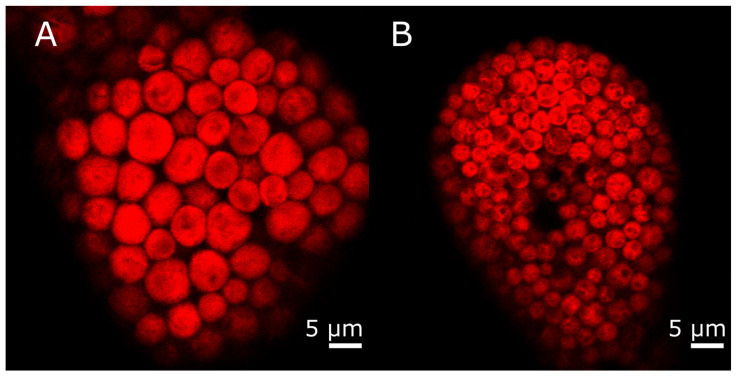
Confocal laser scanning microscope images of kleptoplasts from *Elysia crispata* fed with *Bryopsis* sp. (**A**) or *Acetabularia acetabulum* (**B**). Red coloration indicates chlorophyll *a* fluorescence of the kleptoplasts. Brightness and contrast adjustments were applied to the images to enhance clarity.

In addition to their specific pigment profiles, kleptoplasts from the two groups of sea slugs also exhibited a clear difference in their size and appearance when inspected under a CLSM by detecting chlorophyll fluorescence (Figure 4). Indeed, the average area of an individual kleptoplast in *E. crispata* fed with *Bryopsis* sp. was 28.4 µm^2^ (SD = 8.5, n = 30), whereas the average kleptoplast area of *A. acetabulum* fed sea slugs was significantly smaller, 9.1 µm^2^ (SD = 3.1, n = 30, Welch’s *t*-test, *p* < 0.001).

## 4. Discussion

### 4.1. Rearing of Elysia crispata

The developed protocol allowed the rearing of large numbers of *E. crispata* and the description of its full life cycle under controlled laboratory conditions. Krug et al. [26] observed that most *E. crispata* larvae metamorphosed prior to hatching, in line with our study, but occasionally some larvae hatched as free-swimming veligers, settled after a day and metamorphosed within 2 days after hatching. A significantly longer larval development of up to 35 days and post-hatching metamorphosis within 5 days had been previously reported by Pierce et al. [10]. However, such variability in the timing of different life stages is not unheard of in several invertebrate species displaying lecithotrophic development [9].

Survival was highly variable, with egg masses containing around 1000 embryos yielding an average of 100 juveniles reaching > 2 mm (~10% survival), which were then transferred to LSS. The protocol established critical parameters for *E. crispata* rearing, ensured a consistent supply of specimens (in the order of several hundred per month), clearly securing the needs for cultured specimens for research purposes. It also allowed total control over important features for research, such as age, food source, exposure to a variety of physical and chemical water parameters (e.g., temperature and salinity), as well as previous light history. All these features are key to reducing experimental variability. This standardized approach promotes more reproducible research outputs and makes it possible to advance our scientific understanding of this species’ biology and kleptoplasty in a much more consistent way. In scientific research, maintaining controlled rearing conditions is essential for producing reliable and reproducible results. The establishment of a protocol delineating major developmental timepoints and life cycle duration for *E. crispata* enables researchers worldwide to design and perform experiments without having to rely on the collection of specimens from the wild, which always display high levels of intraspecific variability. To mitigate the reduction in plasticity over time and potential inbreeding-related problems, wild specimens can be occasionally collected or purchased from marine aquarium traders to increase the genetic heterogeneity of the laboratory reared specimens. In our laboratory, wild specimens have been introduced every 1–2 years.

The described protocol, particularly the specific procedures required from egg mass oviposition to hatching and early juvenile development, is somewhat labor-intensive and requires specific equipment (e.g., illuminated temperature-controlled incubator). However, this can be overcome by using inexpensive fish net breeders within the LSS, to which spawned egg masses can be transferred when dark spots are visible within the eggs, about one week post-oviposition. Particular attention should be taken to the mesh size of these nets to prevent the small juveniles to be lost before attaining a larger size. On the other hand, it is also important to guarantee water circulation inside these net breeders, so a compromise in the mesh size must be attained. If clogging occurs, waste products will accumulate and a sharp drop in oxygen levels will occur, ultimately promoting the death of developing sea slugs. Therefore, recently hatched crawling juveniles should be regularly transferred to a new net breeder. This alternative protocol has been successfully employed in periods of less available workforce in the laboratory or when the demand for specimens for research purposes was lower. Nevertheless, despite being less labor-intensive, this rearing protocol was less effective in terms of offspring yield than the original method described above. Hence, the choice between which protocol to use should consider the availability of key equipment and/or workforce, the number of animals needed, the requirements in terms of development stage (embryos, juveniles or adults), and the availability of macroalgae used as food source to maintain sea slugs.

### 4.2. Relevance of Captive Culture for the Study of Kleptoplasty

While the current body of literature on *E. crispata* is relatively limited, there has been a notable increase in research outputs over the past years. This trend suggests a growing scientific interest in this sacoglossan sea slug. Recent research on *E. crispata* encompasses a diverse range of topics, including the study of kleptoplasty [7,23,27], genomics [12], animal behavior and physiology [28], ecology [29], isolation and chemical characterization of natural compounds [14], and assessment of bioactivity [30].

In our study, two thin filamentous macroalgae, *Bryopsis* sp. and *A. acetabulum*, were provided to recently hatched juveniles of *E. crispata*, but these were only able to feed on the former alga. However, it was possible to establish two distinct groups of cultured *E. crispata* harboring different kleptoplasts, as older sea slugs (about 3 months old) were able to shift their diet to *A. acetabulum*. Kleptoplast origin was confirmed by sea slug pigment analysis, matching the pigment signature of the respective algal prey [31,32], with kleptoplasts originating from *Bryopsis* sp. and *A. acetabulum* also being clearly distinguishable via CLSM imaging of sea slugs. Alternatively, or in addition, molecular verification based on DNA barcoding can also be employed to confirm the origin of the kleptoplasts within *E. crispata* specimens that had been raised in the laboratory on *Bryopsis* sp. and later fed on *A. acetabulum* [27].

*Acetabularia acetabulum* is the solitary food and keptoplast source of the long-term retention sacoglossan *Elysia timida* [33]. Photosynthesis-derived carbon from functional *Acetabularia*-derived kleptoplasts was observed in *E. timida* kleptoplast-free tissues, showing translocation of metabolites originating from photosynthesis to different animal cells [34]. Furthermore, the same authors observed a positive impact of kleptoplast photosynthesis on the reproductive output of *E. timida*, confirming the biological relevance of this unique association. On the other hand, its sister species *Elysia cornigera* also feeding upon *A. acetabulum* was unable to maintain long-term functional kleptoplasts [35]. Longevity of *A. acetabulum*-derived kleptoplasts in *E. crispata* was reported to be over twofold that of *B. plumosa* [21]. It seems that both the inherent robustness of the original chloroplasts conjugated with complex mechanisms occurring within the animal host cells contribute to long-term kleptoplasty in photosynthetic sea slugs. The availability of *E. crispata* specimens harboring *A. acetabulum* kleptoplasts opens new experimental options in shedding light over these issues. As the lack of experimental tools and protocols on *Elysia* species is considered a major limitation in the study of kleptoplasty in photosynthetic sea slugs [7], the culture protocol here described may allow researchers to more easily manipulate and assess organellar physiology and function, something not possible to achieve using *E. crispata* collected from the wild.

### 4.3. Elysia crispata Versus Other Kleptoplastic Sea Slugs as a Laboratory Model System

Four long-term retention species within genus *Elysia* can be used as model organisms for the study of kleptoplasty: *E. viridis*, *E. chlorotica*, *E. timida*, and *E. crispata*. Among these sacoglossans, reproductive and developmental strategies vary, influencing the feasibility of their laboratory culture. Both *E. chlorotica* and *E. viridis* display planktotrophic larvae that feed upon microalgae and have a relatively long pelagic development; moreover, they require specific algal cues to trigger metamorphosis, which further constrains their cultivation under controlled conditions [36,37]. A rearing protocol was established for *E. viridis* by Trowbridge [36] and expanded with additional experimental detail by Trowbridge and Todd [38]. However, despite these methodological advances, full laboratory cultivation through the planktotrophic larval stage of *E. viridis* has not been consistently achieved, and most published studies rely on specimens collected from the wild [39,40]. *Elysia chlorotica* has also been successfully reared under laboratory conditions [37]. This unique sea slug, which obtains long-term functional kleptoplasts exclusively from the Xanthophyte alga *Vaucheria litorea*, was the subject of research during the 90s and 00s [41,42], but the small number of experts who studied it have mostly retired or moved on to other subjects. An additional constraint with *E. chlorotica* is the annual die-off reported in wild and laboratory-reared populations, possibly associated with a ubiquitous expression of an endogenous retrovirus [43,44].

To our best knowledge, there are only two kleptoplastic sea slug species, both of them lecithotrophic, that are currently cultured in large numbers in the laboratory over consecutive generations: *E. timida* [33] and *E. crispata* (present study), both having genome sequences available [12,45]. Ideally, both species should be included in a research project to obtain a holistic picture of kleptoplasty, but realistically, research groups often have to select only one of them, as both have specific requirements for their breeding in the laboratory that need to be taken into consideration. Based on our experience, the considerably smaller *E. timida* (adult size 5–15 mm) is far more robust in withstanding less than perfect growth conditions in terms of water quality and temperature fluctuations and can be easily grown in small (e.g., 5–10 L) and inexpensive plastic boxes with minimal aeration [46]. The larger *E. crispata*, on the other hand, produces copious amounts of mucus that quickly fills up a small plastic box, reducing water quality and often leading to the death of stocked animals if the boxes are not cleaned rigorously. For *E. crispata*, it is therefore advisable to grow them in a dedicated LSS with a large volume of water that secures active water cleaning and circulation. As such, the requirements for establishing *E. crispata* cultures can be more costly and time-consuming than for *E. timida*.

The breeding schedules of *E. timida* and *E. crispata* also differ from each other. *Elysia timida* produces large numbers of egg masses throughout the year and its culture can be upscaled in a relatively short period of time, with adults laying eggs within approximately 3 months post-hatching [33]. In contrast, *E. crispata* lays eggs only at specific times, usually at least twice a year, but the exact times when this happens can be difficult to predict. This needs to be considered when planning experiments that require a high number of specimens. However, due to its large size, many experimental procedures will require far fewer *E. crispata* individuals compared to *E. timida*, where tens to hundreds of individuals may need to be sacrificed for a single biochemical analysis. The larger body size of *E. crispata* also allows for a better spatial separation within the animal body in applications such as tissue-specific transcriptomics, that have been performed in the similarly sized sea slug *Plakobranchus ocellatus* [47].

One major disadvantage of *E. timida* is its strict reliance on only one macroalga as food, *A. acetabulum*. While the sea slug itself is relatively easy to maintain, *A. acetabulum* is not; this macroalga grows vegetatively until it reaches the end of its life cycle, by which point it produces gametes and releases them [48]. The vegetative phase cannot be perpetuated by simply cutting the algae to pieces. Instead, rather complex protocols need to be employed to advance the sexual reproduction of *A. acetabulum* [22] and obtain a sufficiently large algal culture that can sustain large numbers of *E. timida*, which will optimistically take 6 to 12 months. This is in stark contrast to *Bryopsis* sp., the main food of *E. crispata* in our cultures; *Bryopsis* sp. grows very fast, and its vegetative growth phase can be maintained simply by cutting out pieces of the algae to inoculate a new culture, as long as necessary. Indeed, even though *E. timida* has many advantages, new research groups interested in studying kleptoplastic sea slugs are advised to choose *E. crispata* over *E. timida* simply due to the ease of growing their algal diet. This is especially important for less experienced researchers in growing macroalgae.

### 4.4. The Marine Aquarium Trade and Species Conservation

The marine aquarium trade is a multimillion-euro global industry, with the EU and the USA being the main importing markets of a multitude of marine species, particularly from tropical coral reefs [49,50]. Nearly all public and private marine aquariums are still stocked with specimens sourced from the wild, with collectors targeting hundreds of reef fish species, as well as corals and other reef-dwelling invertebrates [51]. The captive culture of these species is often considered as a reliable alternative to relieve the current fishing pressure on coral reefs [8,52]. Nonetheless, the remarkable diversity of species traded as marine ornamentals, the lack of knowledge on their early life stages and feeding behavior, as well as multiple technical bottlenecks to successfully achieve their larviculture to metamorphosis, continue to hamper the commercial scale production of most of these species, particularly invertebrates [53].

The sea slug *E. crispata* is commonly sold in the marine aquarium trade because of its ability to eat undesired fast-growing nuisance algae in reef aquariums, including *Bryopsis* spp. A quick search on the internet reveals numerous websites selling this sea slug under different designations, usually referred to as lettuce sea slug due to its parapodia wavy morphology. Interestingly, in some of these websites, *E. crispata* is erroneously referred to as lettuce nudibranch. More importantly, animal origin is usually not specified, although some websites refer the “Caribbean” as a collection site. Most probably, all *E. crispata* specimens sold at these online aquarium suppliers are collected from the wild. In most cases, an “out-of-stock” warning is displayed. When a customer orders lettuce sea slugs, the marine aquarium company contacts local collectors that gather the number of animals required. Retail prices range between EUR 7 and 40 per specimen, with a minimum order of at least 10 to 20 specimens being usually required. It is also important to highlight that mortality rates can be high during handling and shipment of these sea slugs, therefore requiring a larger number of specimens to be collected from the wild to supply demand than that which could be required if such mortality did not occur. Furthermore, *E. crispata* is relatively short-lived, thriving for up to one year under optimal husbandry conditions [54]. If used as a reef aquarium cleaner, hobbyists may require a supply of new sea slugs, hence contributing to increasing the demand for specimens collected from the wild. While from a commercial point of view this may be an advantage for sellers, it certainly results in an increased burden for natural populations of *E. crispata* that may compromise their conservation. As such, the protocol here presented can also contribute to alleviating the current fishing pressure on wild specimens of these sea slugs. Indeed, the protocol developed can easily allow to make available large numbers of captive bred specimens that ship better and are much more resilient to aquarium husbandry conditions than their conspecifics from the wild.

## 5. Conclusions

The development and implementation of standardized protocols for laboratory animal rearing is crucial to ensure animal welfare, scientific research, and sustainable use of marine biological resources. Here, we describe a reliable rearing protocol that resulted in the continuous maintenance of two cultured groups of *E. crispata* harboring different kleptoplasts, originating from the macroalgae *Bryopsis* sp. and *A. acetabulum*. As research interest in *E. crispata* continues to grow, the availability of a standardized rearing protocol will be instrumental to foster high-quality, comparable studies across different laboratories and research groups. Moreover, it can also contribute to relieving the current fishing pressure on the wild populations of this species to supply the marine aquarium trade, therefore actively supporting its conservation.

## Data Availability

Data is contained within the article or Supplementary Material.

## Supplementary Materials

The following supporting information can be downloaded at: https://www.mdpi.com/article/10.3390/biology15020168/s1, Supplementary Figure S1: Flowchart representing key rearing operations in the different development stages of *Elysia crispata*, as represented in Figure 2; Supplementary Figure S2: HPLC chromatograms (440 nm) showing the photosynthetic pigment composition of *E. crispata* fed with the macroalgae *Bryopsis* sp. (A) and *Acetabularia acetabulum* (B); File S1. Raw_Dataset.xlsx.
