# Supplementary material for: Laboratory Rearing of the Photosynthetic Sea Slug Elysia crispata (Gastropoda, Sacoglossa): Implications for the Study of Kleptoplasty and Species Conservation
Source: Biology (Basel). 2026 Jan 17;15(2):168. doi: 10.3390/biology15020168 (PMC12837971; doi:10.3390/biology15020168)
Supplement: Supplementary file 1 [file biology-15-00168-s001.zip › Figure S1.pdf]

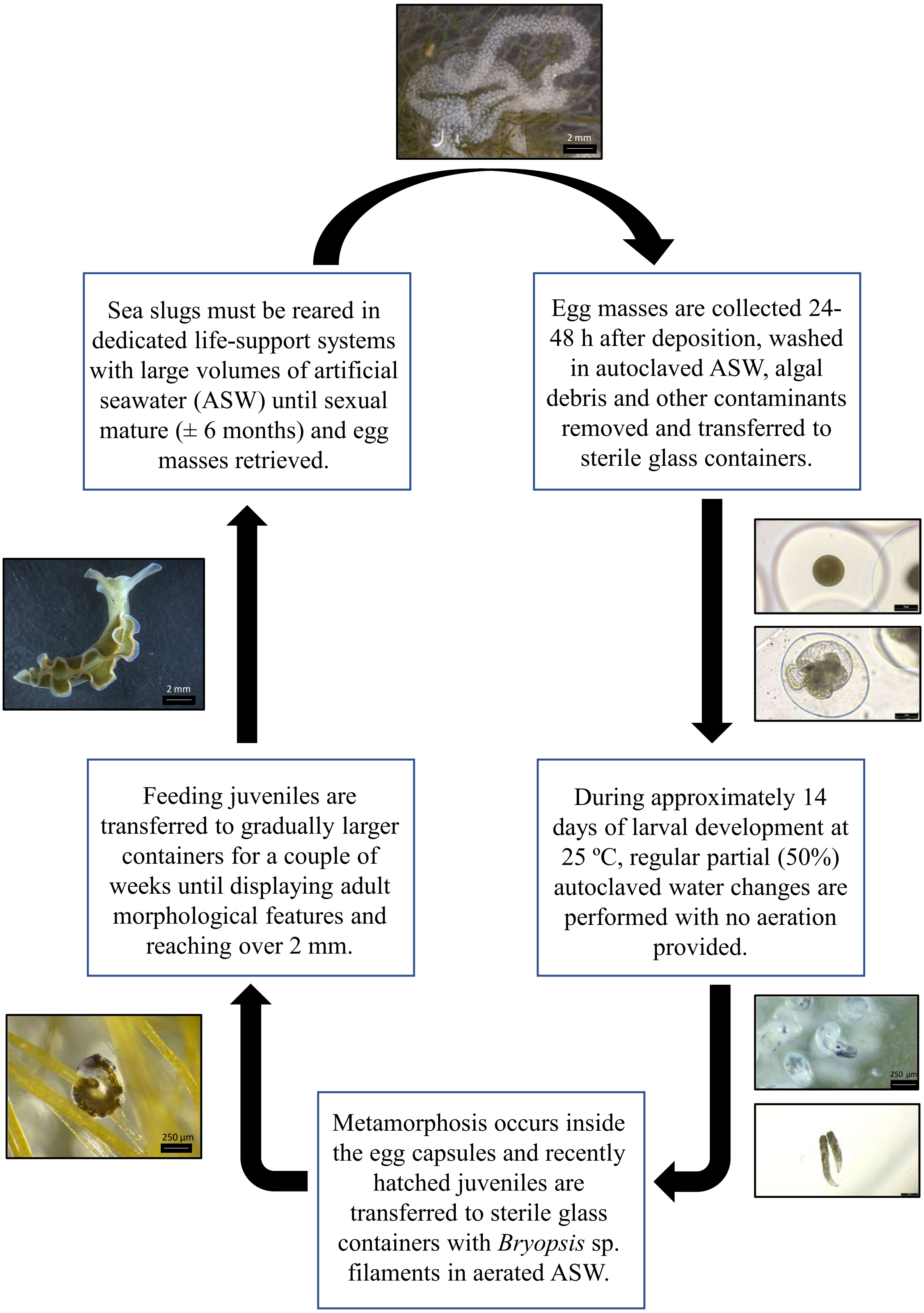


**Figure S1.** Flowchart representing key rearing operations in the different development stages of *Elysia crispata*, as represented in Figure 2.
