# Supplementary material for: Laboratory Rearing of the Photosynthetic Sea Slug Elysia crispata (Gastropoda, Sacoglossa): Implications for the Study of Kleptoplasty and Species Conservation
Source: Biology (Basel). 2026 Jan 17;15(2):168. doi: 10.3390/biology15020168 (PMC12837971; doi:10.3390/biology15020168)
Supplement: Supplementary file 1 [file biology-15-00168-s001.zip › Figure S2.pdf]

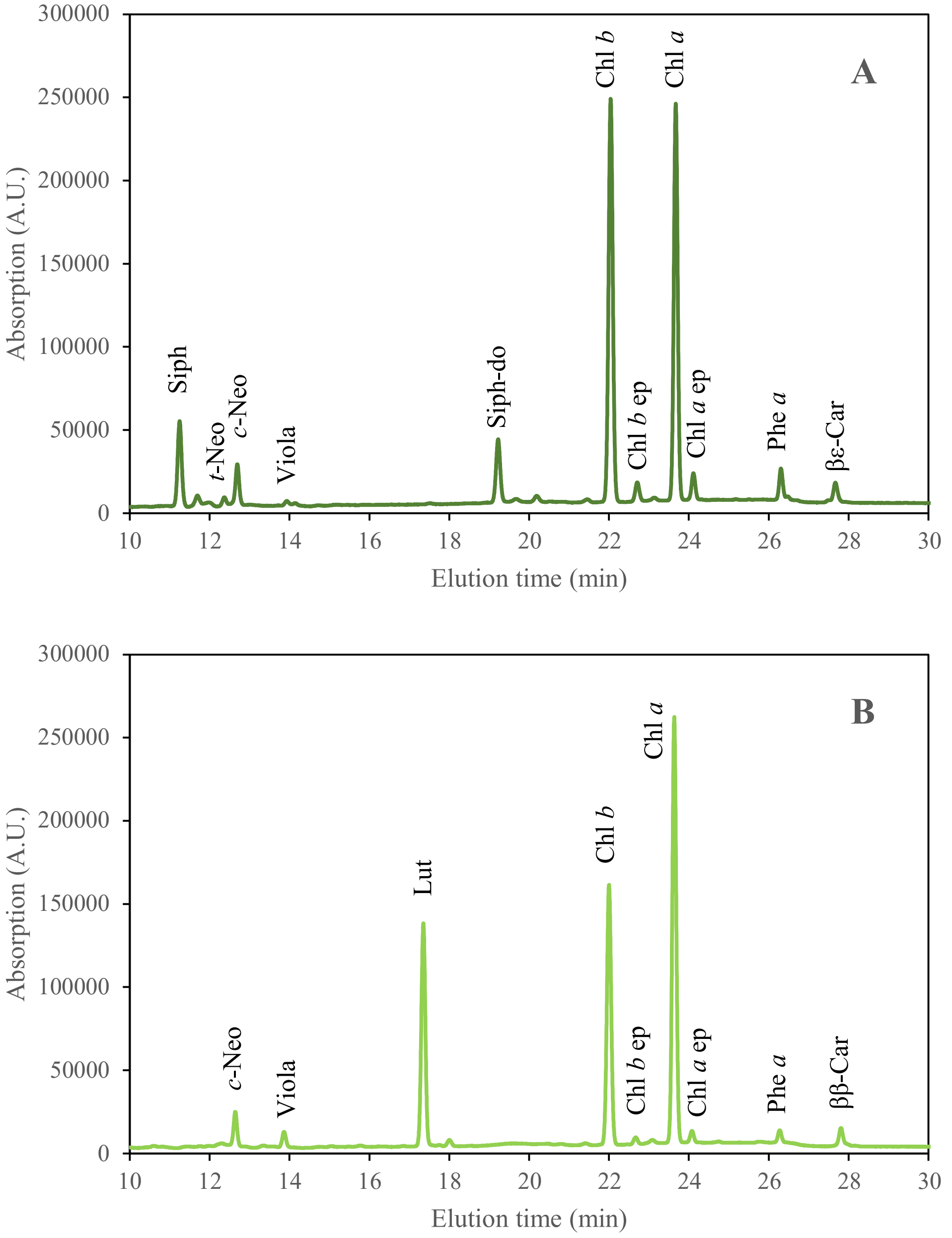

Figure S2. HPLC chromatograms (440 nm) showing the photosynthetic pigment composition of *E. crispata* fed with the macroalgae *Bryopsis* sp. (A) and *Acetabularia acetabulum* (B). Siph: siphonoxanthin; *t*-Neo: *trans*-neoxanthin; *c*-Neo: *cis*-neoxanthin; Viola: violaxanthin; Lut: lutein; Siph-do: siphonaxanthin dodecenoate; Chl *b*: chlorophyll *b*; Chl *b* ep: epimer of chlorophyll *b*; Chl *a*: chlorophyll *a*; Chl *a* ep: epimer of chlorophyll *a*; Phe *a*: pheophytin *a*; βε-Car: β,ε-carotene; ββ-Car: β,β-carotene.
